# Supplementary material for: The PEMDAC phase 2 study of pembrolizumab and entinostat in patients with metastatic uveal melanoma
Source: Nat Commun. 2021 Aug 27;12:5155. doi: 10.1038/s41467-021-25332-w (PMC8397717; doi:10.1038/s41467-021-25332-w)
Supplement: Supplementary file 1 — Supplementary Information [file 41467_2021_25332_MOESM1_ESM.pdf]

**Supplemental information to**

# The PEMDAC phase 2 study of pembrolizumab and entinostat in patients with metastatic uveal melanoma

by L. Ny and H. Jespersen et al.

## Content:

Supplementary Figures. 1-6 and Supplementary questionnaire

## Supplementary Figures

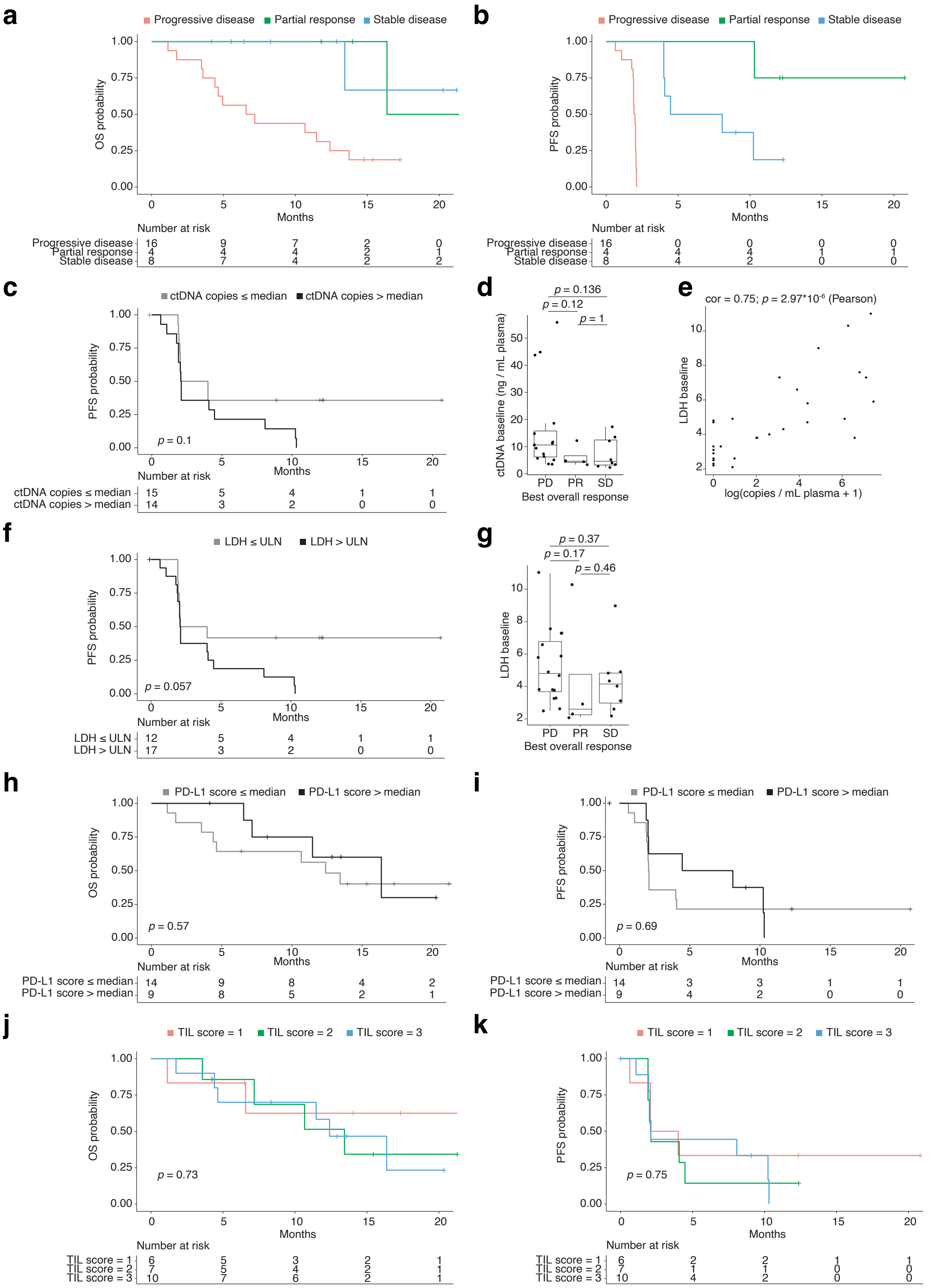


**Supplementary Fig. 1.**
**RECIST, but not PD-L1 or TIL infiltration, impacts survival of patients in the PEMDAC trial. (a)** Overall survival (OS) and **(b)** progression-free survival (PFS) for RECIST response groups in the trial (*n* = 28 patients). **(c)** Kaplan-Meier analysis of PFS for patients with either

high or low baseline ctDNA levels ($n$ = 14 and $n$ = 15 patients, respectively). **(d)** Differences in ctDNA levels at baseline between response groups ($n$ = 16 patients with PD, $n$ = 4 with PR and $n$ = 8 with SD, respectively). Significance was assessed with Wilcoxon rank-sum tests. **(e)** Pearson correlation coefficient and associated $p$-values for association between ctDNA levels and LDH at baseline ($n$ = 29 patients). **(f)** Kaplan-Meier analysis of PFS for patients with higher or lower serum levels of lactate dehydrogenase (LDH) than upper limit of normal (ULN), $n$ = 17 and $n$ = 12, respectively. **(g)** Differences in LDH levels at baseline between response groups ($n$ = 16 patients with PD, $n$ = 4 with PR and $n$ = 8 with SD, respectively), with significance assessed as in (d). **(h-i)** Immunohistochemical analysis of PD-L1 expression was performed and positivity scored (Supplementary Data 4). Patients were dichotomized based on a tumor PD-L1 score greater ($n$ = 9 patients) or not greater ($n$ = 14) than median, and Kaplan-Meier analyses of OS (h) and PFS (i) were performed. **(j-k)** TIL infiltration was assessed in the same histopathological examination as PD-L1 and the tumors ($n$ = 23) were divided into three groups (Supplementary Data 4). The OS (j) and PFS (k) of the patients from the three TIL groups were plotted as Kaplan-Meier curves. PD: progressive disease; PR: partial response; SD: stable disease. In the box plots, horizontal lines indicate median, lower and upper bounds of boxes represent the first and third quartiles, whiskers represent the smallest/largest data point at most 1.5 times inter-quartile range from the lower and upper bound, respectively. In (c), (f) and (h-k), $p$-values for survival associations were calculated using log-rank tests. No adjustments for multiple comparisons were made. All statistical tests were two-sided. Source data are provided as a Source Data file.

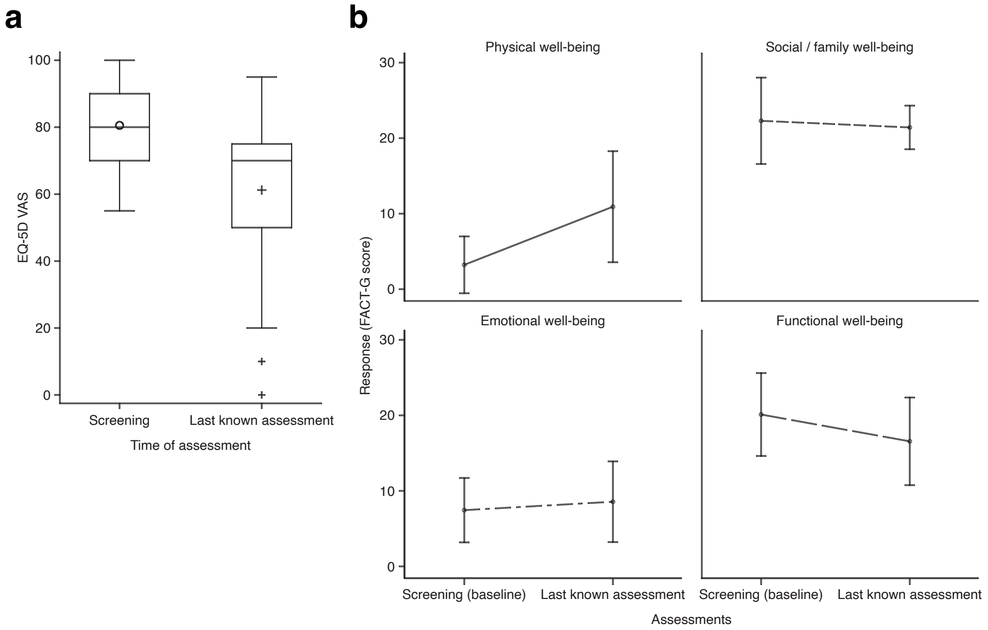


**Supplementary Fig. 2. Quality of life assessments. (a)** Data of visual analog scale (VAS), which records the respondent's self-rated health status on a graduated (0–100). Box plot showing VAS at screening and at last known assessment for $n$ = 27 patients. In the box plots, horizontal lines indicate median, lower and upper bounds of boxes represent the first and third quartiles, whiskers represent the smallest/largest data point at most 1.5 times inter-quartile range from the lower and upper bound, respectively. **(b)** The Functional Assessment of Cancer Therapy – General (FACT-G) score sub-scales at screening and at last known assessments. In the respective categories, $n$ = 27 patients were measured at baseline in each case, whereas the corresponding numbers were $n$ = 25, $n$ = 24, $n$ = 25 and $n$ = 25 patients at last known assessment for the categories "Physical well-being", "Social/family well-being", "Emotional well-being" and "Functional well-being" respectively. Data are mean ± standard deviation.

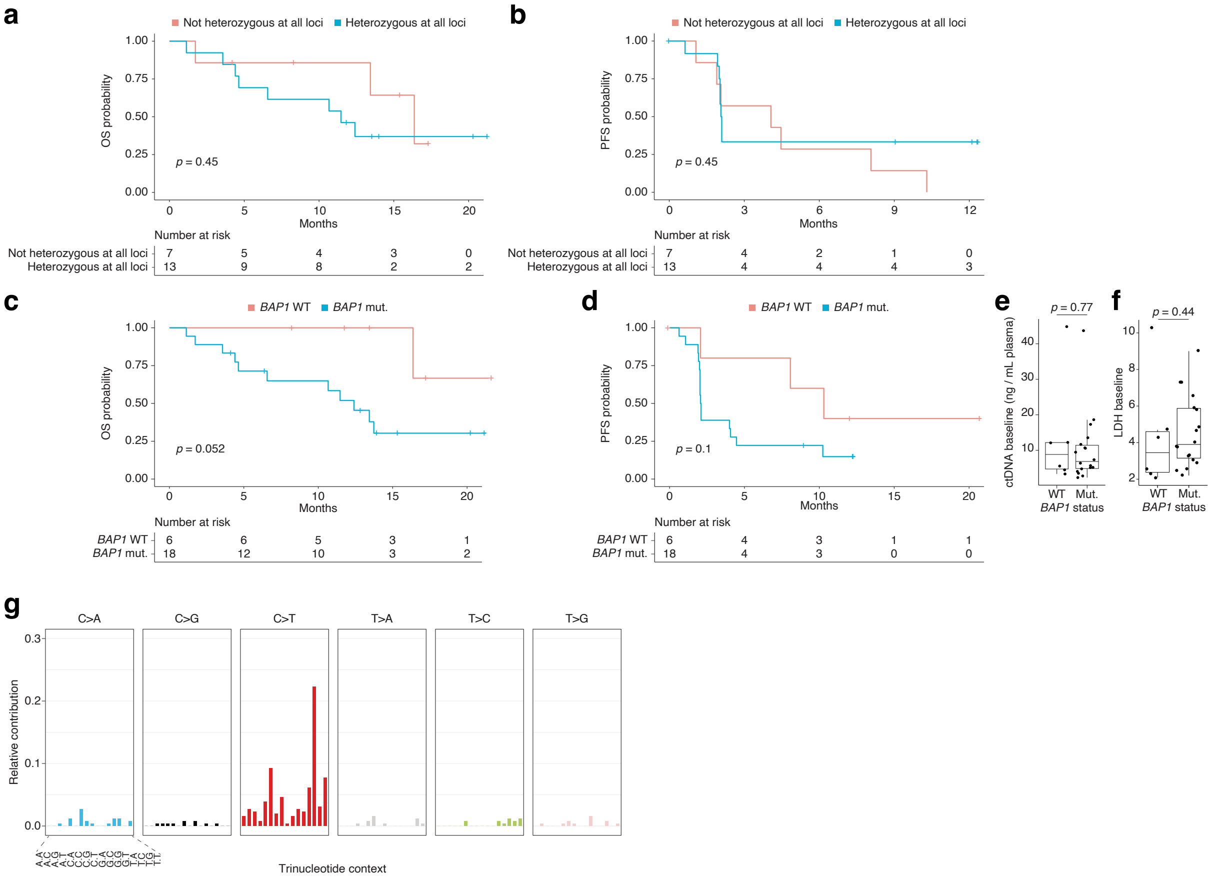


**Supplementary Fig. 3. Genetic and transcriptomic changes related to response. (a)** Overall survival (OS) and **(b)** progression-free survival (PFS) for patients with tumors that have either maximally diverse HLA class I genotypes (i.e six unique alleles) or less. $n = 20$ patients were genotyped and included. $p$-values were calculated with log-rank tests. **(c)** Overall survival and **(d)** progression-free survival for patients with either *BAP1* mutated or wildtype tumors ($n = 18$ and $n = 6$ patients, respectively). In (a-d), $p$-values for survival associations were calculated using log-rank tests. No adjustments for multiple comparisons were made. **(e)** Levels of ctDNA at baseline and **(f)** LDH at baseline compared between *BAP1* mutated and wildtype tumors ($n = 18$ and $n = 6$, respectively). In (e-f), significance was assessed with Wilcoxon rank-sum tests. **(g)** Analysis of both synonymous and non-synonymous mutations in exome sequencing data with respect to COSMIC mutational signatures. Shown is an outlier signature, the UV-associated "signature 7" in 4-022, an iris melanoma sample. All statistical tests were two-sided. Source data are provided as a Source Data file.

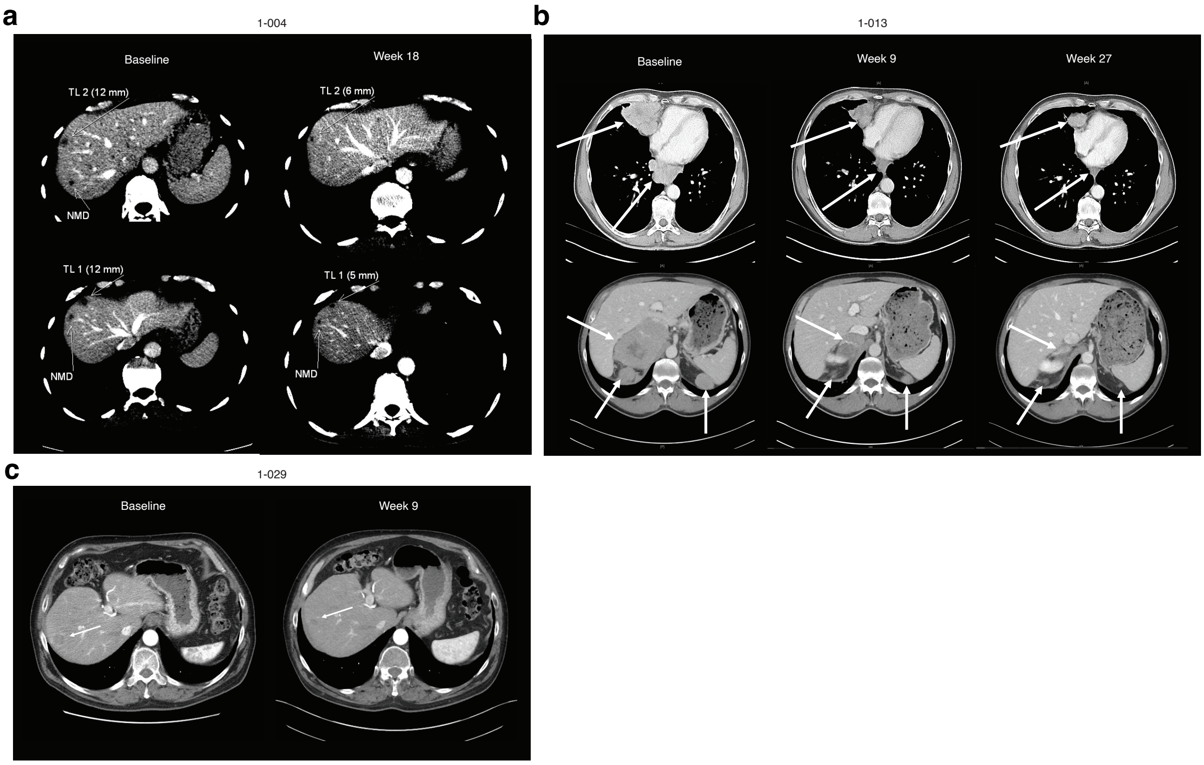


**Supplementary Fig. 4. CT scans of responders. (a)** Patient 1-004, **(b)** 1-013 and **(c)** 1-029, respectively.

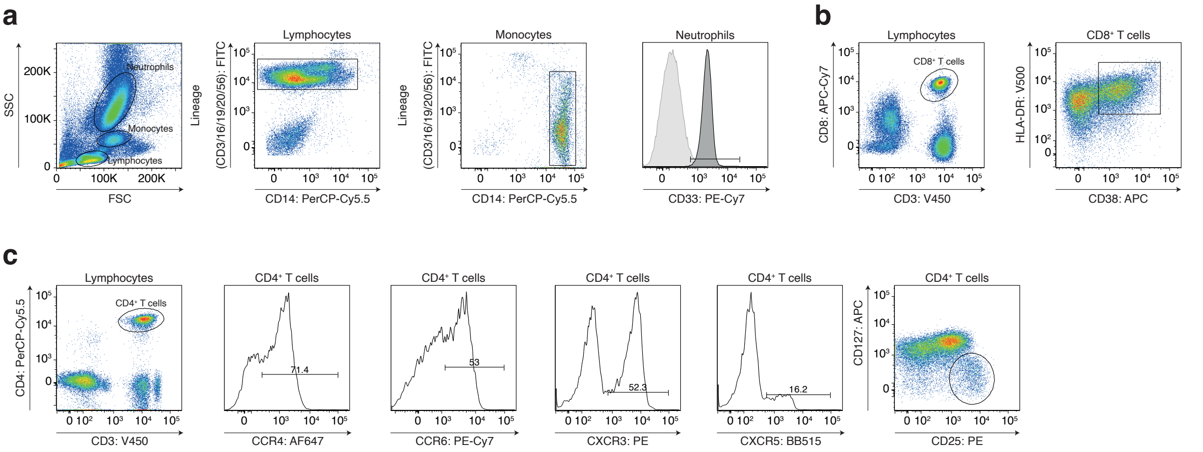


**Supplementary Fig. 5. Gating strategy to identify cell populations from peripheral blood. (a)** Size and complexity gating to identify lymphocytes, monocytes and neutrophils followed by confirmation by lineage staining (CD3, CD16, CD19, CD20, CD56) for lymphocytes, CD14 staining for monocytes and CD33 for neutrophils. **(b)** Identification of CD8$^{+}$ T cells and staining for activation markers HLA-DR and CD38. **(c)** Staining of CD4$^{+}$ T cells to assess expression of surface markers associated with Th2, Th17, Th1, Tfh and Treg cell subsets.

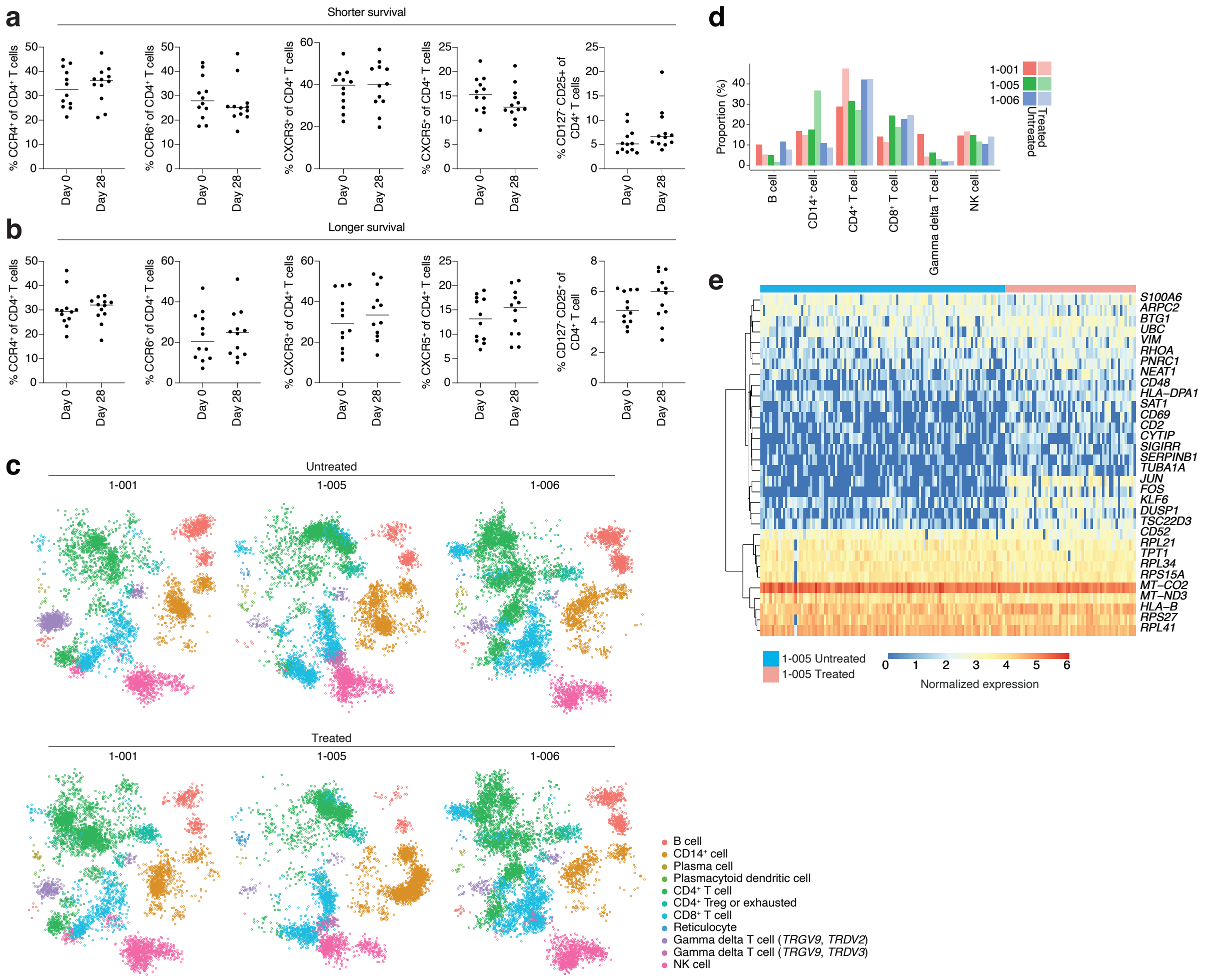


**Supplementary Fig. 6. Expression of cell surfaces markers on circulating CD4$^{+}$ and CD8$^{+}$ T cells following treatment with entinostat and pembrolizumab. (a-b)** Graphs showing frequency of cells expressing CCR4, CCR6, CXCR3, CXCR5, and CD127$^{-}$/CD25$^{+}$ CD4$^{+}$ T cells among patients with shorter ($n$ = 12) (a) and longer ($n$ = 12) overall survival (b) before and after intervention, relative to the median. Statistical analysis was performed using multiple two-sided t-tests and corrected for multiple comparisons using the Holm-Šídák method, where * = $p$<0.05. **(c)** Cell types inferred from 10x Genomics droplet-based single-cell gene expression profiling. **(d)** Proportions of different cell types identified in pre- and post-treatment samples. **(e)** Heatmap of gene expression in T cells of a single clonotype in a sample from patient 1-005 before ($n$ = 108 cells) and after ($n$ = 58 cells) one cycle of treatment with entinostat and pembrolizumab. All statistical tests were two-sided. Source data are provided as a Source Data file.

**Supplementary questionnaire**

| **Secondary Outcome** | **Reported (Y/N)** | **Figures/tables** | **If not reported, please state the reason** |
|---|---|---|---|
| 1. Clinical benefit rate (CBR) [ Time Frame: 18 weeks from first dose ] | Y | In text, abstract and results | |
| 2. Progression free survival (PFS) [ Time Frame: From first dose up to 24 months ] | Y | In text, abstract and results | |
| 3. Overall Survival (OS) [ Time Frame: From first dose up to 24 months ] | Y | In text, abstract and results | |
| 4. Best overall response (BOR) [ Time Frame: From first dose up to 24 months ] | Y | Spider plots and waterfall plots in Figure 1A-B | |
| 5. Time To Response (TTR) [ Time Frame: From first dose up to 24 months ] | Y | Swimmers plot, figure 1C and legend thereof | |
| 6. Duration of objective response (DOR) [ Time Frame: From first dose up to 24 months ] | Y | Swimmers plot, figure 1C and legend thereof | |
| 7. Adverse Events (AEs) and Serious Adverse Events (SAEs). [ Time Frame: From first dose up to 24 months ]<br>Incidence and severity | Y | In text, abstract and results | |
| 8. Eastern Cooperative Oncology Group (ECOG) Performance status (PS) [ Time Frame: 18 weeks from first dose ] | N | In results section | |
| 9. Quality of Life (QoL) assessed by FACT-G [ Time Frame: From first dose up to 24 months ] | N | Supplementary Figure 2 | |
| 10. Quality of Life (QoL) assessed by EQ5D-3L [ Time Frame: From first dose up to 24 months ] | N | Supplementary Figure 2 | |
