# Supplementary material for: The PEMDAC phase 2 study of pembrolizumab and entinostat in patients with metastatic uveal melanoma
Source: Nat Commun. 2021 Aug 27;12:5155. doi: 10.1038/s41467-021-25332-w (PMC8397717; doi:10.1038/s41467-021-25332-w)
Supplement: Supplementary file 3 — Description of Additional Supplementary Files [file 41467_2021_25332_MOESM3_ESM.docx]

Description of Additional Supplementary Files

**Title: Supplementary Data 1**

**Description:** Survival data of patients and biobank registry.

**Title: Supplementary Data 2**

**Description:** Circulating tumor DNA measurements and primers used in the assay.

**Title: Supplementary Data 3**

**Description:**  Detailed statistics.

**Title: Supplementary Data 4**

**Description:** PD-L1 and TIL analysis by IHC.
