# Supplementary material for: The PEMDAC phase 2 study of pembrolizumab and entinostat in patients with metastatic uveal melanoma
Source: Nat Commun. 2021 Aug 27;12:5155. doi: 10.1038/s41467-021-25332-w (PMC8397717; doi:10.1038/s41467-021-25332-w)
Supplement: Supplementary file 8 — Reporting Summary [file 41467_2021_25332_MOESM8_ESM.pdf]

nature research

Corresponding author(s): Jonas Nilsson and Lars Ny

Last updated by author(s): Jul 29, 2021

Reporting Summary

Nature Research wishes to improve the reproducibility of the work that we publish. This form provides structure for consistency and transparency in reporting. For further information on Nature Research policies, see our Editorial Policies and the Editorial Policy Checklist.

Statistics

For all statistical analyses, confirm that the following items are present in the figure legend, table legend, main text, or Methods section.

n/a	Confirmed	
☐	☒	The exact sample size (n) for each experimental group/condition, given as a discrete number and unit of measurement
☐	☒	A statement on whether measurements were taken from distinct samples or whether the same sample was measured repeatedly
☐	☒	The statistical test(s) used AND whether they are one- or two-sided<br>*Only common tests should be described solely by name; describe more complex techniques in the Methods section.*
☐	☒	A description of all covariates tested
☐	☒	A description of any assumptions or corrections, such as tests of normality and adjustment for multiple comparisons
☐	☒	A full description of the statistical parameters including central tendency (e.g. means) or other basic estimates (e.g. regression coefficient) AND variation (e.g. standard deviation) or associated estimates of uncertainty (e.g. confidence intervals)
☐	☒	For null hypothesis testing, the test statistic (e.g. F, t, r) with confidence intervals, effect sizes, degrees of freedom and P value noted<br>*Give P values as exact values whenever suitable.*
☒	☐	For Bayesian analysis, information on the choice of priors and Markov chain Monte Carlo settings
☒	☐	For hierarchical and complex designs, identification of the appropriate level for tests and full reporting of outcomes
☐	☒	Estimates of effect sizes (e.g. Cohen's d, Pearson's r), indicating how they were calculated

Our web collection on statistics for biologists contains articles on many of the points above.

Software and code

Policy information about availability of computer code

Data collection	Flow cytometry: FACS CantoII Biosciences), FACSDiva (v.8.1, BD Biosciences), Debarcer (v. 0.3.1). NGS: NextSeq500
Data analysis	bwa (v. 0.7.17) , GATK (v. 4.1.3.0, includes MarkDuplicates, BaseRecalibrator, ApplyBQSR Mutect 2, FilterMutectCalls, SplitNCigarReads, HaplotypeCaller), samtools (v. 1.9), STAR (V. 2.7.1a), HTSeq (v. 0.11.2), CNVkit (v. 0.9.6), MutationalPatterns (v. 3.0.1), Cell Ranger (v. 3.0.2), Seurat (v. 4.0.3), metacell (v. 0.3.6), DoubletFinder (v. 2.0.3), survival (v. 3.2-11), survminer (v. 0.4.9), R (v. 4.0.5), OptiType (v. 1.3.2), Debarcer (v. 0.3.1), vcf2maf.pl (v. 1.6.17), FlowJo (v. 10.6.1). Custom code used for analysis is available at bitbucket.org/jowkar/pemdac_code.

For manuscripts utilizing custom algorithms or software that are central to the research but not yet described in published literature, software must be made available to editors and reviewers. We strongly encourage code deposition in a community repository (e.g. GitHub). See the Nature Research guidelines for submitting code & software for further information.

Data

Policy information about availability of data

All manuscripts must include a data availability statement. This statement should provide the following information, where applicable:

- Accession codes, unique identifiers, or web links for publicly available datasets
- A list of figures that have associated raw data
- A description of any restrictions on data availability

Sequencing data is available at European Genome-phenome Archive (EGA), accession number EGAS00001005478, under restrictions of controlled access. Figures with associated raw data are Fig. 2 (a-f, h-i), Fig. 3d, Supplementary Fig. 3, Supplementary Fig. 6 (c-e). Online resources and databases used in this study include COSMIC (http://cancer.sanger.ac.uk/cancergenome/assets/signatures_probabilities.txt), GnomAD (https://gnomad.broadinstitute.org/), Encode (http://hgdownload.cse.ucsc.edu/goldenpath/hg19/encodeDCC/wgEncodeMapability/wgEncodeDukeMapabilityRegionsExcludable.bed.gz) and dbSNP (https://www.ncbi.nlm.nih.gov/snp/).

Field-specific reporting

Please select the one below that is the best fit for your research. If you are not sure, read the appropriate sections before making your selection.

[x] Life sciences   [ ] Behavioural & social sciences   [ ] Ecological, evolutionary & environmental sciences

For a reference copy of the document with all sections, see nature.com/documents/nr-reporting-summary-flat.pdf

Life sciences study design

All studies must disclose on these points even when the disclosure is negative.

Sample size	The planned sample size was 29 patients allocated using Simon's optimal two-stage design. At least one confirmed response amongst the first ten patients was required to enroll the additional 19 patients.
Data exclusions	One patient was excluded from the trial due a protocol violation (did not fulfill RECIST criteria for measurable disease) in the first week following the first dose, but still included in survival analyses. RNA and DNA sequencing data from one patient were excluded from analyses due to poor quality.
Replication	Clinical variables were collected in an electronic-CRF database and the study was monitored according to ICH-GCP guidelines. Data extraction and statistical analysis of clinical outcomes were analysed by a professional statistical company, Statistikkonsulterna, as described in the manuscript. Analysis of exploratory outcomes were analysed and re-analysed in order to ensure quality of data.
Randomization	No randomization was applied. Phase 2 study, open label, one treatment arm.
Blinding	There was no blinding.

Reporting for specific materials, systems and methods

We require information from authors about some types of materials, experimental systems and methods used in many studies. Here, indicate whether each material, system or method listed is relevant to your study. If you are not sure if a list item applies to your research, read the appropriate section before selecting a response.

Materials & experimental systems

n/a	Involved in the study	
[ ]	[x]	Antibodies
[x]	[ ]	Eukaryotic cell lines
[x]	[ ]	Palaeontology and archaeology
[x]	[ ]	Animals and other organisms
[ ]	[x]	Human research participants
[ ]	[x]	Clinical data
[x]	[ ]	Dual use research of concern

Methods

n/a	Involved in the study	
[x]	[ ]	ChIP-seq
[ ]	[x]	Flow cytometry
[x]	[ ]	MRI-based neuroimaging

Antibodies

Antibodies used	CCR4-AF647 (BD Biosciences, 557863, 1G1), CCR6-PE-Cy7 (BD Biosciences, 560620, 11A9), CCR7-PE (BD Biosciences, 560765, 150503) CD3-FITC (BD Biosciences, 347663, SK7), CD3-V450 (BD Biosciences, 560365, UCHT1), CD4-PerCP-Cy5.5 (BD Biosciences, 332772, SK3), CD8-APC-Cy7 (BD Biosciences, 560179, SK1), CD11b-APC-Cy7 (BD Biosciences, 557754, ICRF44), CD14-PerCP-Cy5.5 (BD Biosciences, 550787, M5E2), CD16-FITC (BD Biosciences, 555406, 3G8), CD19-FITC (BD Biosciences, 555412, HIB19), CD20-FITC (BD Biosciences, 345792, L27), CD25-PE (BD Biosciences, 341011, 2A3), CD31-FITC (BD Biosciences, 555445, WM59), CD33-PE-Cy7 (BD Biosciences, 333952, P67.6), CD38-APC (BD Biosciences, 345807, HB-7), CD45RA-PE-Cy7 (BD Biosciences, 337186, L48), CD56-FITC (BD Biosciences, 562794, B159), CXCR3-PE (BD Biosciences, 557185, 1C6/CXCR3), CXCR5-BB515 (BD Biosciences, 564624, RF8B2), HLA-DR-V500 (BD Biosciences, 561224, G46-6), CD127-APC (Invitrogen, 17-1278-42, eBioRDR5), PD-L1 (22C3, Merck & Co, MABD122C3).
Validation	Only commercially available antibodies are used. All antibodies have been tested in-house for positive staining using FMOs and/or isotype controls. The anti-PDL1 clone 22C3 is validated for use in clinical diagnostics.

Human research participants

Policy information about studies involving human research participants

Population characteristics	The patients were all patients with metastatic uveal melanoma in four Swedish University Hospitals Feb 2018-Dec 2018 that fullfilled the inclusion and exclusion criteria of the PEMDAC study.
Recruitment	The patients were all patients with metastatic uveal melanoma in four Swedish University Hospitals Feb 2018-Dec 2018 that fullfilled the inclusion and exclusion criteria of the PEMDAC study.
Ethics oversight	The Swedish Ethical Review Authority approved the study (ref no. 692–16)

Note that full information on the approval of the study protocol must also be provided in the manuscript.

Clinical data

Policy information about clinical studies

All manuscripts should comply with the ICMJEguidelines for publication of clinical research and a completedCONSORT checklist must be included with all submissions.

Clinical trial registration	NCT02697630
Study protocol	Jespersen et al., BMC Cancer, 2019. PMID: 31046743.
Data collection	The patients were all patients with metastatic uveal melanoma in four Swedish University Hospitals Feb 2018-Dec 2018 that fullfilled the inclusion and exclusion criteria of the PEMDAC study.
Outcomes	The primary endpoint was objective response rate (ORR) according to RECIST v1.1 criteria. Secondary endpoints included clinical benefit rate (CBR) at week 18, overall survival (OS), progression-free survival (PFS), and incidence and severity of adverse events (AEs). Exploratory endpoints included response by immune-related RECIST (irRECIST) criteria and extensive biomarker analyses.

Flow Cytometry

Plots

Confirm that:

- [x] The axis labels state the marker and fluorochrome used (e.g. CD4-FITC).
- [x] The axis scales are clearly visible. Include numbers along axes only for bottom left plot of group (a 'group' is an analysis of identical markers).
- [x] All plots are contour plots with outliers or pseudocolor plots.
- [x] A numerical value for number of cells or percentage (with statistics) is provided.

Methodology

Sample preparation	Blood samples were analyzed freshly from patients within 24h from the blood was drawn.
Instrument	BD FACS CantoII flow cytometer
Software	FACSDiva software (BD Biosciences) for acquisition and compensation and then analyzed using FlowJo software.
Cell population abundance	At least 1000 events per gate was collected and purity was determined by staining with cell type specific antibodies.
Gating strategy	The major subsets of immune cells in blood (lymphocytes, monocytes and neutrophils) are first identified by conventional gating on size (FSC) and complexity (SSC) and further confirmed by staining for lineage specific markers. From the lymphocyte gate, CD4 and CD8 T cells were identified by co-staining of CD3 to ensure T cell lineage. Among CD4 and CD8 cells, gates indicating positive events were set at the lowest point of overlapping histogram peaks made possible by the bimodal expression pattern or by using a non-expressing population to determine negative events.

- [x] Tick this box to confirm that a figure exemplifying the gating strategy is provided in the Supplementary Information.
